# Epidemiology of pertussis in Alberta, Canada 2004–2015

**DOI:** 10.1186/s12889-017-4468-4

**Published:** 2017-06-02

**Authors:** Xianfang C. Liu, Christopher A. Bell, Kimberley A. Simmonds, Lawrence W. Svenson, Sumana Fathima, Steven J. Drews, Donald P. Schopflocher, Margaret L. Russell

**Affiliations:** 1 Department of Community Health Sciences, University of Calgary, 3280 Hospital Drive NW, Calgary, AB T2N 4Z6 Canada; 2 Alberta Ministry of Health, 17th fl ATB Tower, 10025 Jasper Avenue, Edmonton, Alberta T5J 1S6 Canada; 3 School of Public Health, University of Alberta, Edmonton, AB T6G 1C9 Canada; 4 Division of Preventive Medicine, University of Alberta, Edmonton, AB T6G 2T4 Canada; 5 Provincial Laboratory for Public Health (ProvLab) Calgary Site, Calgary, AB Canada; 6 Provincial Laboratory for Public Health (ProvLab) Edmonton Site, Edmonton, AB Canada; 7 Department of Laboratory Medicine and Pathology, University of Alberta, Edmonton, Alberta T6G 2R3 Canada

**Keywords:** Pertussis, Pertussis vaccine, Epidemiology, Outbreak

## Abstract

**Background:**

We describe the epidemiology of pertussis in Alberta, Canada by person, place, and time between 2004 and 2015, identify outbreak years, and examine vaccination coverage and vaccination timeliness.

**Methods:**

We used health data from Alberta’s Communicable Disease Registry System for the period of January 1, 2004 through August 31, 2015 to identify unique cases of pertussis. Unique cases were deterministically linked to data in Alberta’s immunization repository and health care insurance plan registry. Population estimates and vaccination coverage were extracted from Alberta’s online Interactive Health Data Application. We estimated pertussis incidence rates per 100,000 persons by year, age group, gender, and health zone. Outbreak years were identified using a one-sided cumulative sum (CUSUM) analysis by comparing annual incidence rates to baseline rates.

**Results:**

Over the period, 3510 cases of pertussis were confirmed by laboratory testing or epidemiological linkage. Incidence rates per 100,000 persons were highest in 2004 (20.5), 2005 (13.6), and 2015 (10.4) for all age groups. Incidence rates were highest among the youngest age groups and decreased as age groups increased. Based on CUSUM analysis, 2008 and 2012 met the criteria for outbreak years. Vaccination coverage was over 90% among the general population, however only 61% of cases received at least one dose. About 60% of cases were diagnosed 5+ years after receiving the vaccine. Approximately 87–91% of vaccinated cases did not receive the first three vaccine doses in a timely manner.

**Conclusion:**

Pertussis incidence rates fluctuated over the period across all age groups. The majority of cases had no record of vaccination or were delayed in receiving vaccines. CUSUM analysis was an effective method for identifying outbreaks.

**Electronic supplementary material:**

The online version of this article (doi:10.1186/s12889-017-4468-4) contains supplementary material, which is available to authorized users.

## Background

Pertussis is a vaccine preventable disease that is notifiable, by law, in Alberta, Canada [1]. Pertussis vaccines are included in the Alberta routine childhood immunization schedule with recommended vaccinations scheduled at ages 2, 4, and 6 months, with booster doses at 18 months, 4–6 years, and 14–16 years [2]. Acellular pertussis vaccines are routinely administered as pentavalent vaccine [also immunize against diphtheria, tetanus, poliomyelitis, and *Haemophilus Influenzae* type b (Hib)] for infants and children. A quadrivalent acellular pertussis vaccine (also immunizes against diphtheria, tetanus, and poliomyelitis) is administered at school entry (age 4–6 years) and a trivalent acellular vaccine (immunizes against diphtheria, tetanus, pertussis) at ages 14–16 years [3]. In 1997, all whole cell pertussis vaccines were replaced by acellular vaccines [3].

There has been a resurgence of pertussis cases in Alberta in the last 15 years among all age groups, despite high vaccination coverage [4]. Theories to explain this resurgence include: waning immunity, low vaccine effectiveness, inadequate immune response, lack of boosting, or changes in the disease agent [5–7]. Pertussis outbreaks occur cyclically and are expected every 2–5 years [8].

Ongoing surveillance is important to monitor disease and detect outbreaks. We describe the epidemiology of pertussis disease by time, person, and place in Alberta over the period 2004–2015. We also examine pertussis incidence rates to identify outbreak years and explore for differences in vaccination coverage, duration of vaccine protection, and vaccination timeliness.

## Methods

### Pertussis case definition

The Alberta Health (AH) pertussis case definition for confirmed cases requires: i) laboratory confirmation, which includes isolation of *Bordetella pertussis* or detection of *B. pertussis* nucleic acid using polymerase chain reaction (PCR) from a clinical specimen; or ii) epidemiological linkage to laboratory confirmed cases, plus compatible cough illness with no other known cause [1]. Cases that are epidemiologically linked to laboratory confirmed cases are those that have had contact, and transmission of the agent by the usual modes of transmission is plausible. The Provincial Laboratory for Public Health (ProvLab) conducts laboratory testing for all *B. pertussis* cases in Alberta. Clinical specimens are collected by nasopharyngeal swab and sent to ProvLab for *B. pertussis* testing using real-time PCR [9]. Specimens test positive for *B. pertussis* if the crossing point occurs at ≤35 cycles [10].

### Data sources and analysis

Pertussis reporting is required by law under the Public Health Act [1]. Pertussis reports are captured in the Communicable Disease Reporting System (CDRS), which is a passive surveillance system in Alberta [11]. Alberta’s Immunization and Adverse Reaction to Immunization (Imm/ARI) repository records individual vaccination histories for all publicly funded vaccines. Imm/ARI has complete province-wide coverage records since 2006 [12]. Patient registration files for Alberta’s universal, publicly funded healthcare system are recorded in the Alberta Health Care Insurance Plan (AHCIP) Central Stakeholder Registry. Registration with the AHCIP is mandatory for all residents of the province. AHCIP contains demographic details on all registrants, including a personal health number that acts as a unique personal identifier (ULI), which permits linkage of individual records across administrative health databases.

For the period of January 1, 2004 through August 31, 2015, we extracted CDRS data to capture the number of people classified with confirmed pertussis and diagnoses dates. We used administrative data in Imm/ARI and AHCIP to capture immunization dates, numbers of doses of pertussis containing vaccine received, as well as client information including gender, age, and health zone of residence at time of diagnosis. We used the ULI to deterministically link health records from CDRS, Imm/ARI, and AHCIP, at the individual level. Data linkage, extraction, and anonymization were performed by AH employees (data custodians) prior to release to the researchers.

Alberta population estimates and vaccination coverage were extracted from the online Interactive Health Data Application (IHDA) database [4]. We estimated incidence rates by year of diagnosis, age group, gender, and health zone over the period 2004–2015. Incidence rates were calculated for each characteristic as number of cases per 100,000 persons per year. Data were analyzed using SAS 9.3 (SAS Institute Inc., Cary, NC 2011) and graphed in SigmaPlot 13.5 (Systat Software Inc.).

### Outbreak years CUSUM analysis

An outbreak for a given population, time, or location has been defined as an increase in the occurrence of pertussis disease where the measured values exceeded the baseline occurrence in previous years [1]. We identified outbreak years by comparing annual incidence rates to baseline rates using a one-sided cumulative sum (CUSUM) analysis. The one-sided CUSUM analysis estimates whether positive increases in annual incidence rates exceed baseline incidence rates based on set criteria [13].

The one-sided CUSUM analysis was plotted in a chart based on the following equation: C_0_ = 0, C_t_ = max[0,C_t_-_1_ + (y_t_-μ_t_-k)] [13]. The initial CUSUM value C_0_ = 0, C_t_ was the CUSUM value at time t, y_t_ was the incidence rate at time t, μ_t_ was the baseline incidence rate at time t, and k was the reference value constant. Baseline incidence rates were calculated as the average of incidence rates in previous years and the current year. The reference value k and threshold value h were chosen based on the criteria for identifying an outbreak. These values can be adjusted to change the sensitivity of the analysis. For our analysis, k = 0.5 and h = 3.

If the CUSUM value exceeded the threshold value h, then the incidence rate exceeded the baseline rate, and the criteria for an outbreak were met. Incidence rates that were close to baseline will have CUSUM values close to 0. After the CUSUM value exceeds the threshold value, the CUSUM value and baseline rates are reset and the process continues.

### Vaccination coverage, duration of vaccine protection, and vaccination timeliness

Pertussis vaccination coverage data in IHDA were estimated as the proportion of children aged 2 years who received one or more doses of pertussis vaccine between 2004 and 2015 [4]. While IHDA estimated vaccination coverage data from 2004 to 2007 for one or more doses of vaccine, coverage data from 2008 to 2015 were dose-specific and estimated for each of the first four doses. Additionally, in 2008, the Alberta government reorganized the nine regional health authorities into a single health service provider with five health zones (Calgary, Central, Edmonton, North, South). As a result, vaccination coverage data extracted from IHDA for the periods 2004–2007 and 2008–2015 were estimated for different numbers of doses and health zones.

Among cases, we estimated vaccination coverage, duration of vaccine protection, and vaccination timeliness. Duration of vaccine protection was calculated as the amount of time between the last vaccination prior to disease diagnosis. We categorized duration of vaccine protection into weeks, months, and years. Vaccination timeliness was calculated as the proportion of the cases that received the appropriate number of doses at the recommended ages. The first three doses are recommended at ages 2, 4, and 6 months and three booster doses at ages 18 months, 4–6 years, and grade 9 (age 14–16 years). Cases who received the appropriate dose within 1 month after the recommended age were considered timely, otherwise they were considered delayed if they received the dose more than 1 month after the recommended age.

## Results

From January 1, 2004 through August 31, 2015, 3510 people were diagnosed with pertussis. Of these, 2675 (76.2%) were lab confirmed cases and 835 (23.8%) epidemiologically linked cases. Numbers of cases were highest in 2004, 2005, 2014, and 2015 (Table 1). Of the cases, 403 (11.5%) were aged <1 year, 576 (16.4%) were aged 1–4 years, 539 (15.4%) were aged 5–9 years, 798 (22.7%) were aged 10–14 years, 308 (8.8%) were aged 15–19 years, and 886 (25.2%) were aged 20 years or older (Table 1). There were 1882 females (53.6%) and 1627 males (46.4%). Over the period, 946 cases resided in the North zone, 815 in Central, 751 in Edmonton, 406 in South, and 357 in Calgary (Table 1). Detailed incidence rate data for year of diagnosis, age group, gender, and health zone is provided in Additional file 1.

**Table 1:** Descriptive characteristics of pertussis cases

		Number of cases (%)
Total number of cases		3510 (100)
Year of diagnosis	2004	664 (18.9)
	2005	452 (12.9)
	2006	211 (6.0)
	2007	128 (3.6)
	2008	239 (6.8)
	2009	193 (5.5)
	2010	68 (1.9)
	2011	114 (3.2)
	2012	326 (9.3)
	2013	302 (8.6)
	2014	378 (10.8)
	2015	435 (12.4)
Age groups (years)	<1	403 (11.5)
	1–4	576 (16.4)
	5–9	539 (15.4)
	10–14	798 (22.7)
	15–19	308 (8.8)
	20+	886 (25.2)
Gender	Female	1882 (53.6)
	Male	1627 (46.4)
	Unknown	1 (0)
Health zone	Calgary	357 (10.2)
	Central	815 (23.2)
	Edmonton	751 (21.4)
	North	946 (27.0)
	South	406 (11.6)
	N/A^a^	235 (6.7)
Vaccination histories among cases:		
Never vaccinated		1370 (39.0)
Vaccinated after disease diagnosis		515 (14.7)
Vaccinated out-of-province		324 (9.2)
Number of people vaccinated in Alberta before diagnosis		1301 (37.1)
Number of doses received by people vaccinated in Alberta prior to disease	1	131 (10.0% of those vaccinated)
	2	53 (4.1)
	3	130 (10.0)
	4	247 (19.0)
	5+	740 (56.9)
Duration of vaccine protection	2 weeks to 1 month	42 (3.2)
	1–2 months	25 (1.9)
	2–3 months	19 (1.5)
	3 months to 1 year	94 (7.2)
	1–3 years	206 (15.8)
	3–5 years	131 (10.1)
	5+ years	784 (60.3)
Timeliness (Number of cases vaccinated at specified ages for recommended doses)	2 months	982 (75.5% of vaccinated cases)
	4 months	809 (65.4)
	6 months	703 (58.8)
	18 months	473 (41.6)
	4–6 years	755 (80.7)
	14–16 years	317 (71.2)

^a^N/A: health zone information was missing

Although the number of cases aged <1 year remained stable, the proportion of cases aged <1 year decreased between 2007 and 2015. Approximately 23% of all cases diagnosed in 2007 were aged <1 year. By 2015, less than 9% of all cases were aged <1 year (i.e., there was a shift in the proportion of cases towards older age groups). In 2007, 15% of cases were aged 1–4 years, 17% aged 5–9 years, and 13% aged 10–14 years. By 2015, 25% were aged 1–4 years, 20% aged 5–9 years, and 19% aged 10–14 years.

### Pertussis incidence rates

**Fig. 1:**
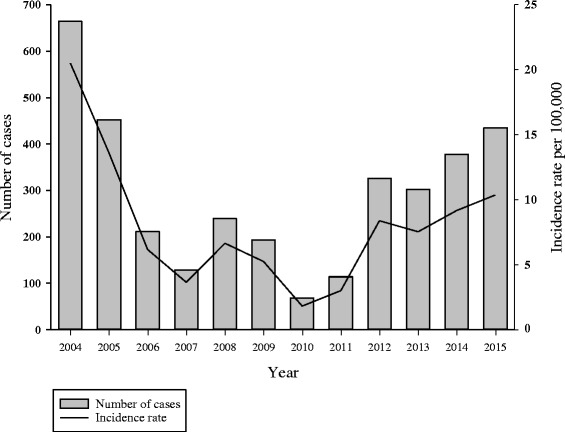
Number of pertussis cases and incidence rate by year

Over the period, the annual pertussis incidence rate ranged from 1.8 to 20.5 cases per 100,000 persons. Incidence rates per 100,000 persons were highest in 2004 (20.5), 2005 (13.6), 2014 (9.2), and 2015 (10.4) (Fig. 1). Incidence rates declined between 2004 and 2010 and increased between 2010 and 2015. Pertussis incidence rates per 100,000 persons reached a maximum in 2008 (6.6) and 2012 (8.4).

**Fig. 2:**
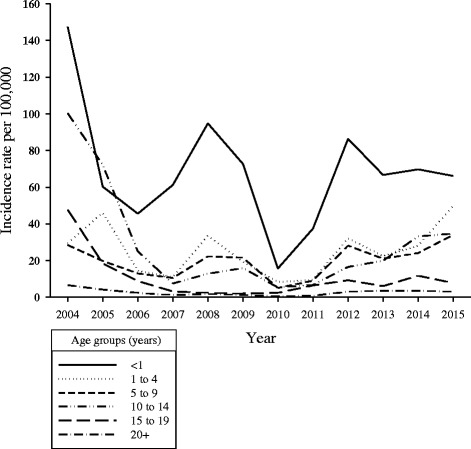
Annual pertussis incidence rate by age groups

Incidence rates were generally higher among younger age groups and decreased as age groups increased (Fig. 2) over the period. Infants aged <1 year had the highest incidence rates per 100,000 (147.6 in 2004 and 66.1 in 2015) over the period, except in 2005 where those aged 10–14 had the highest rate (72.1). Incidence rates peaked every 3–4 years across all age groups. Since 2010, incidence rates have been trending upwards among those aged 1–4, 5–9, and 10–14 years. Incidence rates by gender were similar between females and males over the period.

Incidence rates per 100,000 by health zone were highest in the Central (183), North (215), and South (143) zones and lowest in the Calgary (26) and Edmonton (64) zones over the period. While a large number of cases occurred in Calgary and Edmonton (Table 1), these zones have a population size approximately three to five times larger than other zones (average population estimates over the period: 1.38 and 1.16 million in Calgary and Edmonton vs 0.28 to 0.44 million in other zones).

### CUSUM analysis

**Fig. 3:**
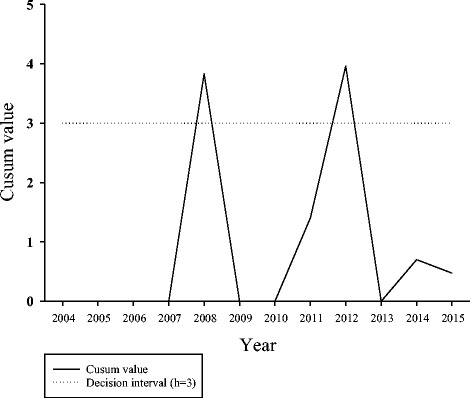
One-sided CUSUM chart for annual incidence rates

Based on CUSUM analysis, 2008 and 2012 were identified as outbreak years (Fig. 3). CUSUM values in 2008 and 2012 exceeded the threshold value indicating that incidence rates were higher than baseline. Although incidence rates in 2014 and 2015 were also high, the CUSUM value did not exceed the threshold value and thus the outbreak criterion was not met. Incidence rates in other years were similar to baseline.

### Vaccination coverage

Alberta vaccination coverage rates among 2 year olds who completed all four doses ranged between 80 and 84% between 2004 and 2007. Between 2008 and 2015, vaccination coverage for each of the first three doses was high; approximately 90–95% of the target population was vaccinated with one to three doses of vaccine by age 2, and 75.7% was vaccinated for the fourth dose (Table 2). Annual vaccination coverage in the population decreased slightly for each dose over the period 2008–2015. Vaccination coverage declined from around 96.5% to 94.7% for the first dose, 94.9 to 92.1 for the second dose, 92.9 to 89.7 for the third dose, and 78.8 to 77 for the booster [4].

**Table 2:** Province-level vaccination population coverage (by age 2 years) in Alberta 2008–2015 by dose number

Dose number	Vaccination coverage
1	95.2%
2	93.0%
3	90.5%
4	75.7%

Vaccination coverage among cases was 37.1% (1301 vaccinated with ≥1 doses of pertussis vaccine in Alberta prior to disease diagnosis). Other cases were either vaccinated after disease diagnosis (14.7%), not vaccinated (39%), or received vaccines out-of-province (9.2%) (Table 1). There were 403 cases aged less than 1 year. Of these cases, 157 (39%) were aged <3 months, 90 (22%) aged 3–5 months, and 156 (39%) aged 6 months to less than 1 year. Of the 403 cases, 244 (60.5%) had received 1 or more doses of vaccine. Of the 244, 96 (39%) were aged <3 months, 64 (26%) were aged 3–5 months, and 84 (34%) were aged 6 months to less than 1 year.

### Duration of vaccine protection

Of the 1301 cases that were vaccinated in Alberta prior to disease, 131 (10%) had received only one vaccine dose, 53 (4.1%) received two doses, 130 (10%) received three doses, 247 (19%) four doses, and 740 (56.9%) five or more doses. The majority of cases were diagnosed five or more years after vaccination (60.3%), while a smaller proportion were diagnosed within a year (13.8%), 1–3 years (15.8%), or within 3–5 years (10.1%) (Table 1). Of the 740 cases that had received five or more doses, 555 (75%) were diagnosed five or more years after vaccination (data not shown).

### Vaccination timeliness among cases

Among vaccinated cases (*n* = 1301), 982 (75.5% of vaccinated cases) received the first dose at age 2 months. Of the 1237 cases who received two or more doses, 809 (65.4%) received the vaccine at 4 months. Of the 1196 who received three doses, 703 (58.8%) received the vaccine at 6 months. For booster doses, 473 cases (41.6%) received the first booster at age 18 months, 755 (80.7%) received the second booster at ages 4–6 years, and 317 (71.2%) received the third booster at ages 14–16 years.

## Discussion

This study described the epidemiology of pertussis in Alberta from 2004 to 2015, and examined vaccination coverage and vaccination timeliness. All notifiable pertussis cases in Alberta, Canada were identified from the CDRS surveillance system. Cases included those which were confirmed PCR positive or through epidemiological linkage to a lab confirmed case. Data linkage with Imm/ARI and AHCIP allowed us to describe vaccination coverage and timeliness among cases, while IHDA data provided a complete picture of coverage in Alberta.

Our findings were consistent with observations across Canada that pertussis incidence rates have increased in recent years [8]. Incidence rates in Canada have declined between 2004 (10 cases per 100,000) and 2011 (2 cases per 100,000) but greatly increased in 2012 to 13.9 per 100,000. This sudden increase has been observed in several Canadian provinces and territories (British Columbia, Alberta, Manitoba, Ontario, Quebec, New Brunswick, and Yukon) [8]. The age distribution of cases in Alberta were consistent with CDC results that show the largest proportion of pertussis cases was among people aged 10–19 years from 1990 to 2015 in the United States [14–16]. From 2001 to 2003, 33% of cases were aged 10–19 years [14–16]. From 2011 to 2015, 28% to 33.8% of cases were aged 11–19 years [17–20]. These effects may be due to waning natural and vaccine-induced immunity over time and vaccination programs targeting younger populations.

Immunity to pertussis starts to wane 3–5 years after the last vaccine dose and individuals will become more susceptible after 5–8 years [6, 7]. Our findings are consistent with this, and show that most people were diagnosed with pertussis 5+ years after vaccination. Waning immunity may also be related to the increasing proportion of cases in older age groups in Alberta. However, these results could also be explained by differences in vaccination coverage, incomplete/partial vaccination, and types of vaccine received.

Our findings were consistent with another study conducted in Alberta which observed a large number of lab confirmed sporadic pertussis cases in 2004, 2005, 2008, and 2012 [10]. Although Fathima and colleagues [10] identified six community-based outbreaks (COBs) in 2004, they also identified three COBs in 2005 and 2009, and four COBs in 2011 [10]. COBs were determined based on cases localized within a community and number of cases varied depending on the size of the community and past pertussis activity. For the purpose of our study, we identified outbreaks at the provincial level using both lab confirmed and epidemiologically linked cases. Thus, while both studies observed similar trends in the number of pertussis cases, differences in our definition and measurement of outbreaks accounted for differences in the number of outbreaks identified.

Despite high vaccination coverage in the general population, there has been an increase in the incidence rate of pertussis in the past few years. Although the majority of cases were not vaccinated, those that were vaccinated had received the appropriate vaccine doses in a timely manner. The observed vaccination coverage and timeliness among cases may be related to accessibility to healthcare and adherence to vaccination guidelines. Indeed, the Central, North, and South zones had lower vaccination coverage than Calgary and Edmonton for all doses. Not surprisingly, these three zones also had higher pertussis incidence rates compared to Calgary and Edmonton. Low measles vaccination rates among children have been observed in southern Alberta, and could be explained by low vaccination rates in private schools within specific cultural/religious communities and by including vaccination rates among home-schooled in community-level estimates [21]. These factors may be related to low pertussis vaccination rates in southern Alberta. Promotion to increase adherence to childhood vaccinations may be necessary to encourage those who are likely to have incomplete/partial vaccinations.

Although incidence rates in 2004 and 2015 were high, we could not determine whether these were outbreak years due to lack of data. However, based on historical pertussis incidence rates from 2000 to 2004 [1], it is likely that 2004 would have met the outbreak criteria in CUSUM analysis. Similarly, if pertussis cases diagnosed after August 31, 2015 were included in the analysis, it is likely that 2015 would also be an outbreak year.

The CUSUM analysis is an effective method for identifying outbreaks by comparing incidence rates to expected baseline rate. The CUSUM analysis is sensitive to detecting small deviations from the baseline and is appropriate for analyzing surveillance data when the CUSUM value is reset after it exceeds the threshold value [22]. This method is especially useful for early detection of outbreaks [23], and also for continuous monitoring of data collected over different time intervals and across different age groups, gender, and health zones.

Our study describes the epidemiology of pertussis by using several administrative data sources. One strength of this study was the use of a passive surveillance system (CDRS) to capture all pertussis cases for over a decade. Secondly, Alberta’s electronic health system allows cases to be linked to other data registries to assess laboratory results, vaccination and health status, i.e. by using a unique lifetime identification number that all Alberta residents will have. Thirdly, ProvLab has done all lab testing for pertussis in the province since 2004, and thus all laboratory confirmed cases were captured in this study. Finally, linking several data sources allowed us to perform CUSUM analysis to estimate outbreaks in the province. This study does come with some limitations. Cases that are not reported will be missed and not captured by the passive surveillance system. Another limitation was that we did not capture the vaccination histories of individuals, who were vaccinated out-of-province. Thus, we could not determine whether they received vaccines at the recommended ages. These individuals were included in the analysis for vaccination coverage but excluded from the analysis for timeliness.

## Conclusion

The ongoing collection of data could be used to evaluate vaccination programs, assess disease impact, identify outbreaks, support prevention activities, and disseminate communicable disease information to health professionals and the general public in a timely fashion. This allows for immediate public health actions in response to changes in disease activity. Although pertussis vaccination coverage remained high, annual pertussis incidence rates in Alberta fluctuated among age groups and health zones between 2004 and 2015. CUSUM analysis identified outbreaks in 2008 and 2012, when incidence rates exceeded baseline rates. The majority of cases had no record of vaccination or were delayed in receiving vaccines.

## Additional file

Additional file 1: Table S1. Numbers of Pertussis cases and (incidence rates per 100,000) by year of diagnosis, 2004–2015. **Table S2.**Numbers of Pertussis cases and (incidence rates per 100,000) by year and age group, 2004–2015. **Table S3.**Numbers of Pertussis cases and (incidence rates per 100,000) by year and gender, 2004-2015. **Table S4.**Numbers of Pertussis cases and (incidence rates per 100,000) by year and health zone, 2004-2015. (DOCX 15 kb)

## Abbreviations

AH: Alberta Health
AHCIP: Alberta health care insurance plan central stakeholder registry
CDRS: Communicable disease reporting system
COB: Community-based outbreak
CUSUM: Cumulative sum
Hib: *Haemophilus Influenzae* type b
IHDA: Interactive health data application
Imm/ARI: Immunization and adverse reaction to immunization repository
PCR: Polymerase chain reaction
ProvLab: Provincial laboratory for public health
ULI: Unique lifetime identifier
