# Supplementary material for: Epidemiology of pertussis in Alberta, Canada 2004–2015
Source: BMC Public Health. 2017 Jun 2;17:539. doi: 10.1186/s12889-017-4468-4 (PMC5457605; doi:10.1186/s12889-017-4468-4)
Supplement: Additional file 1: Table S1. — Numbers of Pertussis cases and (incidence rates per 100,000) by year of diagnosis, 2004–2015. Table S2.Numbers of Pertussis cases and (incidence rates per 100,000) by year and age group, 2004–2015. Table S3.Numbers of Pertussis cases and (incidence rates per 100,000) by year and gender, 2004-2015. Table S4.Numbers of Pertussis cases and (incidence rates per 100,000) by year and health zone, 2004-2015. (DOCX 15 kb) [file 12889_2017_4468_MOESM1_ESM.docx]

Table S1: Numbers of Pertussis cases and (incidence rates per 100,000) by year of diagnosis, 2004-2015

| Year | Number of cases | Incidence rate per 100,000 |
| --- | --- | --- |
| 2004 | 664 | 20.5 |
| 2005 | 452 | 13.6 |
| 2006 | 211 | 6.2 |
| 2007 | 128 | 3.6 |
| 2008 | 239 | 6.7 |
| 2009 | 193 | 5.3 |
| 2010 | 68 | 1.8 |
| 2011 | 114 | 3.0 |
| 2012 | 326 | 8.4 |
| 2013 | 302 | 7.5 |
| 2014 | 378 | 9.2 |
| 2015 | 435 | 10.4 |

Table S2: Numbers of Pertussis cases and (incidence rates per 100,000) by year and age group, 2004-2015

| Year | Age groups (years) | | | | | |
| --- | --- | --- | --- | --- | --- | --- |
|  | <1 | 1 to 4 | 5 to 9 | 10 to 14 | 15 to 19 | 20+ |
| 2004 | 60 cases (147.6) | 46 (29.5) | 59 (28.5) | 229 (100.4) | 114 (47.7) | 156 (6.6) |
| 2005 | 25 (60.3) | 74 (46.1) | 41 (19.8) | 164 (72.1) | 45 (18.4) | 103 (4.2) |
| 2006 | 20 (45.6) | 24 (14.4) | 27 (13.0) | 57 (25.0) | 22 (8.9) | 61 (2.4) |
| 2007 | 29 (61.3) | 19 (11.1) | 22 (10.5) | 17 (7.5) | 8 (3.2) | 33 (1.23) |
| 2008 | 47 (94.8) | 60 (33.6) | 47 (22.3) | 29 (12.9) | 6 (2.4) | 50 (1.9) |
| 2009 | 37 (72.7) | 36 (19.4) | 46 (21.6) | 36 (16.0) | 5 (2.0) | 33 (1.2) |
| 2010 | 8 (15.7) | 16 (8.3) | 11 (5.1) | 12 (5.4) | 6 (2.4) | 15 (0.5) |
| 2011 | 19 (37.5) | 19 (9.5) | 20 (9.0) | 15 (6.7) | 16 (6.5) | 25 (0.9) |
| 2012 | 45 (86.4) | 66 (32.2) | 65 (28.1) | 37 (16.6) | 23 (9.3) | 90 (3.1) |
| 2013 | 36 (66.7) | 47 (22.5) | 51 (21.1) | 45 (20.0) | 15 (6.1) | 108 (3.6) |
| 2014 | 39 (69.8) | 60 (28.1) | 61 (24.2) | 76 (33.2) | 29 (11.8) | 113 (3.6) |
| 2015 | 38 (66.1) | 109 (49.8) | 89 (34.0) | 81 (34.8) | 19 (7.8) | 99 (3.1) |

Table S3: Numbers of Pertussis cases and (incidence rates per 100,000) by year and gender, 2004-2015

|  | Gender | |
| --- | --- | --- |
| Year | Female | Male |
| 2004 | 354 cases (22.1) | 310 (18.9) |
| 2005 | 245 (15.0) | 207 (12.3) |
| 2006 | 102 (6.1) | 109 (6.3) |
| 2007 | 69 (4.0) | 59 (3.3) |
| 2008 | 128 (7.3) | 111 (6.1) |
| 2009 | 103 (5.7) | 90 (4.8) |
| 2010 | 31 (1.7) | 37 (2.0) |
| 2011 | 60 (3.2) | 54 (2.8) |
| 2012 | 170 (8.9) | 156 (7.9) |
| 2013 | 171 (8.7) | 131 (6.4) |
| 2014 | 208 (10.3) | 170 (8.1) |
| 2015 | 238 (11.5) | 197 (9.2) |

Table S4: Numbers of Pertussis cases and (incidence rates per 100,000) by year and health zone, 2004-2015

|  | Health Zone* | | | | |
| --- | --- | --- | --- | --- | --- |
| Year | CALGARY | CENTRAL | EDMONTON | NORTH | SOUTH |
| 2004 | 29 cases (2.5) | 236 (57.9) | 133 (13.2) | 159 (40.8) | 46 (17.8) |
| 2005 | 46 (3.8) | 85 (20.4) | 91 (8.8) | 150 (37.7) | 61 (23.1) |
| 2006 | 63 (5.0) | 43 (10.1) | 42 (3.9) | 49 (12.0) | 4 (1.5) |
| 2007 | 23 (1.8) | 35 (8.0) | 35 (3.2) | 31 (7.4) | 2 (0.7) |
| 2008 | 12 (0.9) | 22 (5.0) | 31 (2.8) | 155 (36.3) | 2 (0.7) |
| 2009 | 28 (2.1) | 20 (4.5) | 43 (3.7) | 5 (1.2) | 84 (29.6) |
| 2010 | 6 (0.4) | 13 (2.9) | 10 (0.9) | 23 (5.2) | 3 (1.0) |
| 2011 | 4 (0.3) | 25 (5.5) | 18 (1.5) | 55 (12.3) | 1 (0.4) |
| 2012 | 34 (2.3) | 51 (11.1) | 128 (10.5) | 43 (9.4) | 60 (20.5) |
| 2013 | 46 (3.1) | 78 (16.7) | 57 (4.5) | 58 (12.2) | 49 (16.6) |
| 2014 | 37 (2.4) | 134 (28.3) | 95 (7.3) | 41 (8.5) | 49 (16.3) |
| 2015 | 29 (1.8) | 73 (15.3) | 68 (5.1) | 177 (36.1) | 45 (14.9) |

*Note: No health zone information for 235 cases
